# Stress, Allostase und Organversagen: Zum 90. Jahrestag der Erstbeschreibung des „allgemeinen Anpassungssyndroms“

**DOI:** 10.1007/s00101-026-01735-6

**Published:** 2026-09-24

**Authors:** Michael Bauer, Reinhard Wetzker

**Affiliations:** Klinik für Anästhesiologie und Intensivmedizin, Universitätsklinikum Jena, Am Klinikum 1, 07747 Jena, Deutschland

**Keywords:** Anpassungsreaktion, Intensivmedizin, Resilienz, Hormese, Sepsis, Adaptive response, Intensive Care Medicine, Resilience, Hormesis, Sepsis

## Abstract

Vor 90 Jahren erschien in *Nature* eine bahnbrechende Arbeit des Endokrinologen Hans Selye, in der er eine umfassende Theorie des allgemeinen Anpassungssyndroms („general adaptation syndrome“) von Organismen auf schädigende Reize entwickelte. Selye beobachtete an Ratten, die er unterschiedlichen physikalischen oder chemischen Noxen unterwarf, stereotype hormonelle Reaktionen und führte in späteren Arbeiten den heute ubiquitär verwendeten Begriff „Stress“ hierfür ein. Dabei entlehnte er den Begriff aus der Physik („stress and strain“), wo er die Verformung eines Festkörpers durch einwirkende Kräfte beschreibt. Selye räumte später ein, dass es im biomedizinischen Kontext besser gewesen wäre, statt der auslösenden Faktoren „Stress“ die Belastungs*effekte* – also „strain“ – zur Beschreibung des allgemeinen Anpassungssyndroms zu nutzen. Analog zum Überdehnen eines Festkörpers bis zum Bruch, wird beim Überschreiten der Reparatur- und Defensivsysteme lebender Organismen ein „point of no return“ erreicht, der durch Organschäden bis hin zum Tod charakterisiert ist. Selyes Forschung hat grundlegend zum Verständnis der Schädigungsreaktionen gerade auch in der Intensivmedizin beigetragen. So lassen sich z. B. traumainduzierte Stresseffekte in chirurgischen Patienten durch Analgesie und Sedierung modulieren. In der vorliegenden Übersicht sollen die Fortschritte in der medizinischen Stressforschung, insbesondere das Konzept der Allostase und der Hormese sowie deren zugrunde liegenden molekularen Mechanismen für das ärztliche Handeln am Beispiel des Organversagens bei Sepsis erläutert werden.

## Abstract (en)

A groundbreaking paper by the endocrinologist Hans Selye was published in *Nature* 90 years ago in which he developed a comprehensive theory of the g adaptation syndrome in organisms responding to harmful stimuli. Selye observed stereotypical hormonal reactions in rats subjected to a wide variety of physical and chemical irritants and later on introduced the now ubiquitous term “stress” to describe these phenomena. He borrowed the term from physics (stress and strain), where it describes the deformation of a solid body by applied forces. Selye later conceded that in a biomedical context, it would have been more accurate to use the *effects* of stress, i.e., strain, to describe the general adaptation syndrome rather than the triggering factors. Analogous to overstretching a solid until it fractures, exceeding the repair and defence systems of living organisms leads to a point of no return, characterized by organ damage and ultimately death. Selye’s research has fundamentally contributed to our understanding of injury responses particularly in intensive care medicine. For example, trauma-induced stress effects in surgical patients can be successfully modulated through analgesia and sedation. This review aims to explain the advances in medical stress research, particularly the concepts of allostasis and hormesis and their underlying molecular mechanisms for medical actions, using the paradigm of organ failure in sepsis as an example.

## Einleitung

„Stress“ ist heute ein gebräuchlicher Begriff der Alltagssprache und geht auf das Konzept einer stereotypen Anpassungsreaktion auf unterschiedlichste schädigende Ereignisse zurück. Das Konzept wurde 1936 durch den aus Österreich-Ungarn stammenden Mediziner Hans Selye (Infobox) erstmals formuliert und beschreibt eine Gruppe von stereotypen psychischen und physischen Anpassungsreaktionen, die durch Reize (Stressoren) ausgelöst werden und dann den Organismus zur Bewältigung besonderer Anforderungen befähigen [27]. Selyes Arbeit in der Zeitschrift *Nature* von 1936 gilt als Ausgangspunkt der modernen Stressforschung; der Begriff Stress wurde durch Selyes spätere Arbeiten in die biomedizinische Terminologie eingeführt und verbreitet [28]. Selye nutzte den Begriff Stress, der ursprünglich aus der Festkörperphysik entstammt, um in ihren Auswirkungen ähnliche Reaktionen des Organismus auf äußere und innere Bedrohungen zu beschreiben [29]. Auf dem Wissensstand seiner Zeit erkannte Selye, dass Traumen, Vergiftungen, Verbrennungen, aber auch psychische Belastungen verwandte hormongesteuerte Reaktionen im Organismus auslösen. Alle von ihm untersuchten „Stressereignisse“ bewirkten über die Ausschüttung von Stresshormonen wie Adrenalin oder Cortisol ähnliche Anpassungsreaktionen, die zunächst adaptiv, später maladaptiv wirkten (in der Festkörperphysik „stress and strain“ [18]). Hans Selye erkannte auch, dass die Stressanpassung und -bewältigung eine zentrale Überlebensfrage für alle Organismen darstellt.

Selyes Grundvorstellungen über Stress als Anpassungsprozess an äußere Reize und Belastungen (Stressoren) wurden in den letzten Jahrzehnten weiterentwickelt und sind zentral für die Anästhesiologie, insbesondere die Intensivmedizin am Paradebeispiel des Organversagens.

Zur semiquantitativen Beschreibung der Anpassungsfähigkeit von höheren Organismen entwickelte im Weiteren der amerikanische Neurobiologe Peter Sterling am Ende des 20. Jahrhunderts den Begriff der *Allostase* als adaptiven Prozess, der die Erhaltung der Stabilität des Organismus in Anwesenheit von Stressoren ermöglicht. Hierbei erweiterte er insbesondere den Begriff Homöostase dahingehend, dass unterschiedliche physiologische Systeme, wie Säure-Basen-Haushalt oder Metabolismus und Immunität durch Stress induzierte Abweichungen von der Norm unterschiedlich gut tolerieren. Sterling definierte auf dieser Grundlage Gesundheit als optimale Allostase und Krankheit über unzureichende Anpassungsfähigkeit [36].

Adaptive Stressreaktionen von Organismen sind in besonderer Weise zeitlich bestimmt. Mechanistisch unterscheiden lassen sich kurzfristige (Sekunden, Minuten, Stunden), mittelfristige (Stunden, Tage, Monate) und langfristige (Jahre/Generationen) Anpassungsreaktionen. Auf molekularer Ebene fußen kurzfristige Anpassungsreaktionen auf Signaltransduktions- und Expressionsprozessen von Mediatoren, mittelfristige Anpassungsreaktionen beziehen epigenetische Mechanismen ein, während langfristige Reaktionen genetisch bestimmt sind. Eine Besonderheit mittelfristiger Anpassungsreaktionen ist die Entwicklung eines Stressgedächtnisses, welches eine schnelle effektive Anpassung an wiederkehrende Stressereignisse gestattet und damit im darwinistischen Sinne einen Selektionsvorteil ermöglicht [25].

Der vorliegende Beitrag beschreibt die grundlegenden Mechanismen von kurz- und mittelfristigen Stressreaktionen und beleuchtet ihre Rolle in der Anästhesie und Intensivmedizin. Zentraler Schwerpunkt sind dabei die übergreifenden Mechanismen der Dynamik von Stressreaktionen und die Abhängigkeit dieser Reaktionen von der Intensität Stress-auslösender Vorgänge, den Stressoren. Dabei soll am Beispiel der perioperativen Stressantwort die zentrale Bedeutung von Stressreaktionen für die Entwicklung einer Organdysfunktion in Anästhesie und Intensivmedizin im Kontext von Selyes Konzept beschrieben werden.

## Die akute Stressantwort: Angst, Kampf und Fluchtreaktion

In einer – extrem seltenen – belastungs- und stressfreien Lage befindet sich ein Organismus energetisch im Grundzustand der Homöostase. Die Energiezufuhr durch Ernährung und Atmen dient nur zur Aufrechterhaltung basaler Funktionen von Zellen und Organen; insbesondere können Wachstum, Proliferation und Fortpflanzung als für den Erhalt der Spezies bedeutsame Reaktionen priorisiert werden [40].

**Abb. 1:**
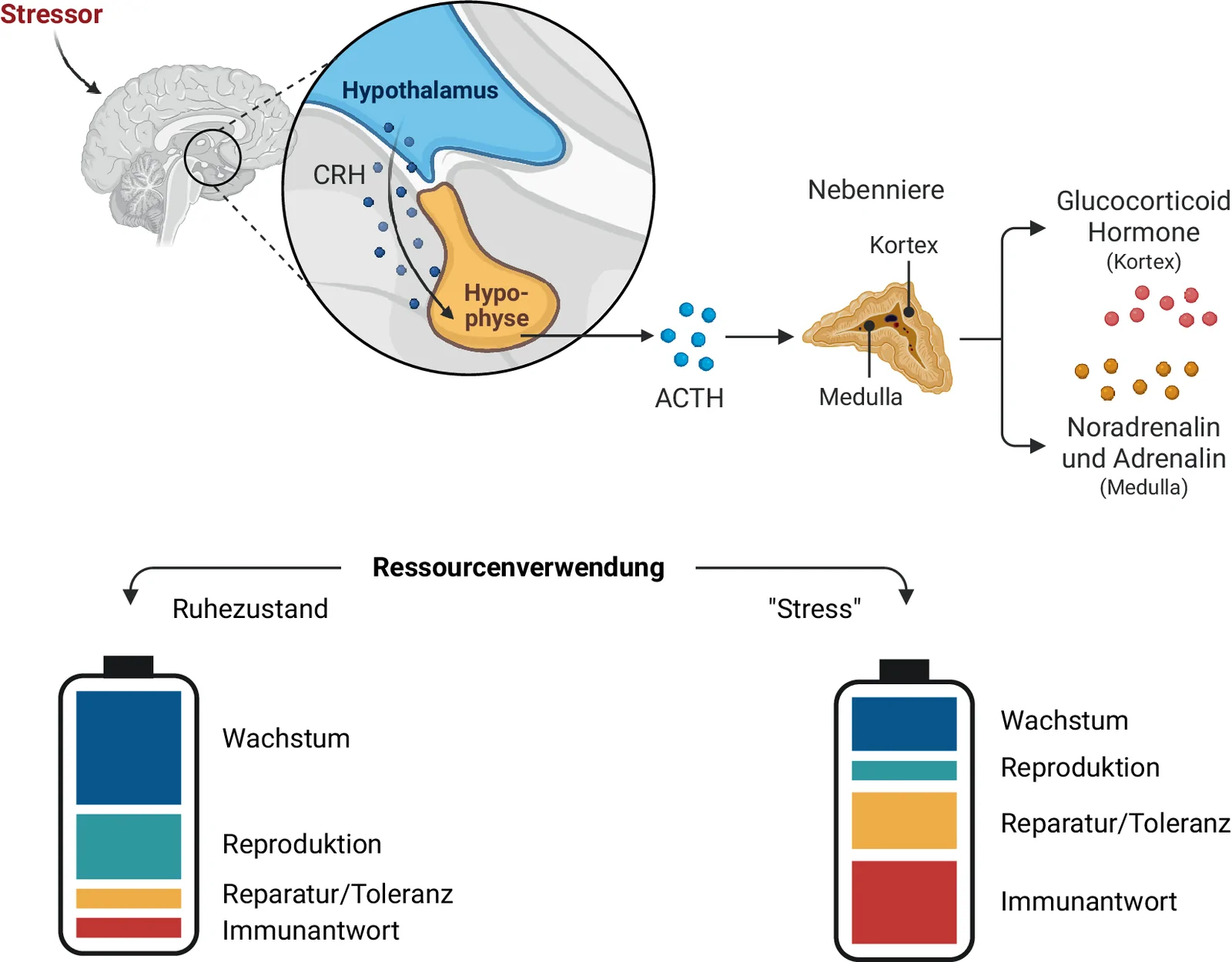
Stressantwort – Konzept der induzierbaren Abwehr auf Stressereignisse. Als Folge eines Stressereignisses mit Aktivierung der Hypothalamus-Hypophysen-Nebennierenachse werden basale Funktionen von Zellen und Organen, wie Wachstum, Proliferation und Fortpflanzung, zu Gunsten von Abwehr- und Reparaturprozessen gedrosselt. Klinisches Korrelat ist beispielsweise die Amenorrhö bei unterernährten Frauen und kritisch kranken Patientinnen.

Treffen starke Stressoren auf Organismen, so lösen sie ein quasireflektorisches Reaktionsmuster von „Angst‑, Kampf- und Fluchtreaktionen“ aus, die darauf zielen, die Lebensfunktionen zu erhalten und das Überleben zu ermöglichen. Dies erfordert eine Neuausrichtung des Energiestoffwechsels von Erhaltungsfunktionen auf Defensiv- und Reparaturfunktionen [40]. Hierzu werden in höheren Lebewesen durch über die Sinnesorgane eingehende Alarmsignale, Anpassungsreaktionen aktiviert und, z. B. die Energiebereitstellung für die Muskulatur erhöht (Abb. 1). Selye hat erstmalig die Redundanzen dieser Stressreaktionen beobachtet und ihren Zusammenhang mit der hypothalamisch-hypophysären Regulation der Nebennierenaktivierung beschrieben [27]. Völlig unterschiedliche Stressoren bewirken dabei ähnliche Reaktionsmuster, die sowohl adaptiven als auch maladaptiven Stressreaktionen zugrunde liegen [31].

Charakteristisch für Stressreaktionen ist eine Reaktionskette, bestehend aus Sensoren, Mediatoren und Effektoren: Typische Sensoren sind im Menschen die Sinnesorgane, die über das Großhirn die Ausschüttung von Stresshormonen als Mediatoren auslösen [36]. Dabei wird in einer schnellen neuronalen Reaktion zuerst Adrenalin freigesetzt. Zudem werden, neuronal gesteuert, Kortikoide über die Hypothalamus-Hypophysen-Achse in der Nebennierenrinde ausgeschüttet (Abb. 1). Beide Stresshormone lösen zunächst über Effektorzellen und -organe eine Erhöhung der Widerstandsfähigkeit (Resilienz) des Organismus gegenüber dem Stressor aus. So wird über die stresshormonvermittelte Aktivierung des Herz-Kreislauf-Systems z. B. die Energieversorgung der Skelettmuskulatur gesteigert. Der Körper ist nun bereit zur „Fight-or-flight“-Reaktion [7].

**Abb. 2:**
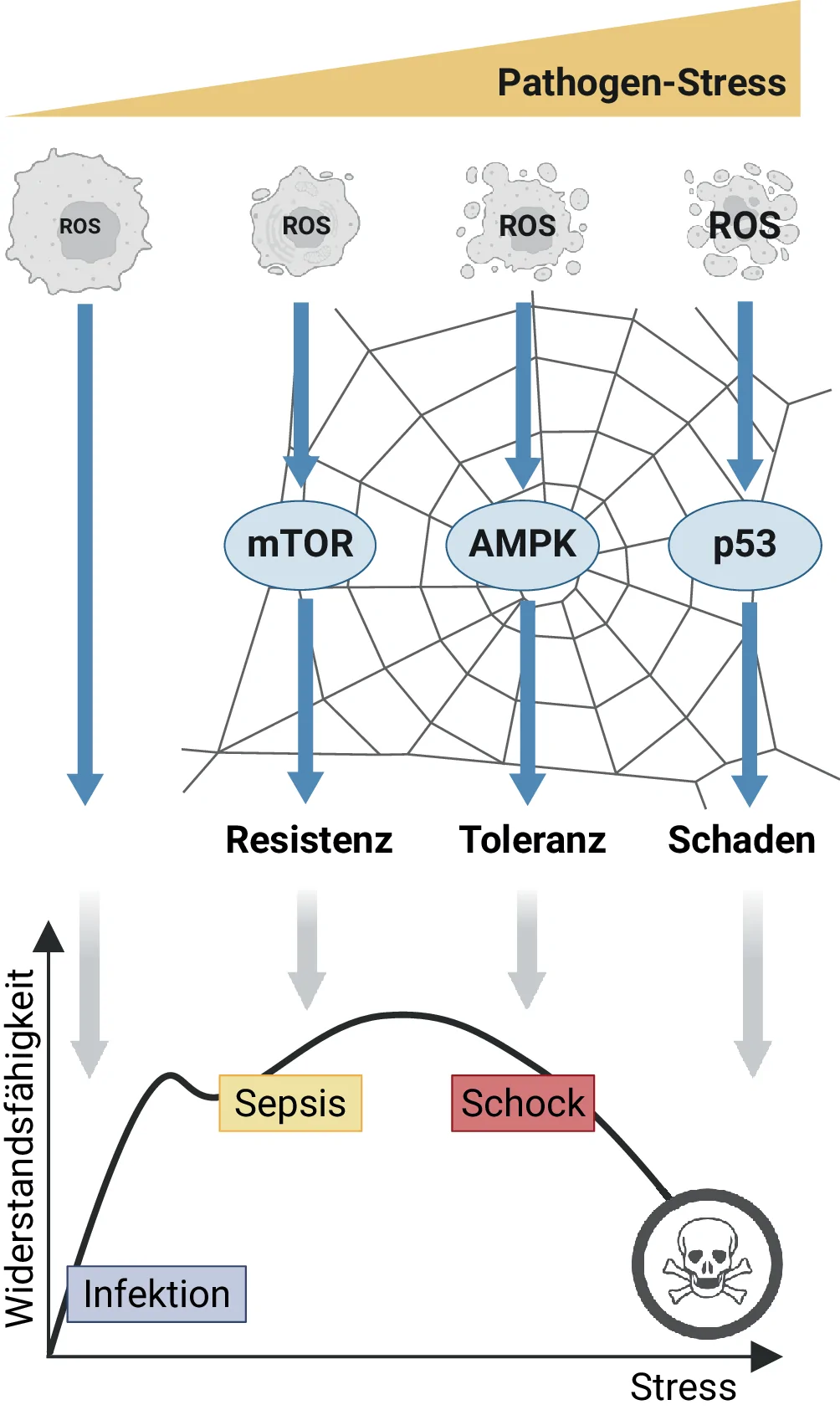
Adaptive und maladaptive Antworten des Organismus auf Pathogen-Stress und die zugrunde liegenden Signalphänomene. In Abhängigkeit von der Schwere des Insults werden in den betroffenen Zellen zunehmende Konzentrationen von Stressmediatoren, wie z. B. *ROS* (reactive oxygene species) generiert. Die *ROS* aktivieren dosisabhängig ein Netzwerk eng verzahnter Signalwege. Das Signalprotein *mTOR* (mechanistic Target of Rapamycin) wirkt dabei als Schlüsselmediator anaboler Resistenzreaktionen, während *AMPK* (AMP-stimulierbare Kinase) überwiegend katabole Reparatur- und Toleranzreaktionen reguliert. Über das Signalprotein *p53* wird bei hohem Pathogen-Stress und hohen *ROS*-Konzentrationen der Zelltod eingeleitet. Alle genannten Signalwege tragen zu den adaptiven und maladaptiven Anpassungsreaktionen im Sinne der Grundvorstellungen von Selye am Beispiel der Sepsis bei.

Wesentliche Fortschritte konnten in den letzten 90 Jahren bezüglich des Verständnisses der zellulär zugrunde liegenden Signaltransduktionsmechanismen erzielt werden. Insbesondere ist ein Gleichgewicht der intrazellulären Signalmediatoren mTOR (mechanistic Target of Rapamycin) und AMPK (AMP-stimulierbare Kinase), welches in der Zelle als Sensor für den Energiezustand dient und die energetischen Erfordernisse um Stressreaktionen im Kontext anaboler als auch kataboler Prozesse steuert, für die Stressantwort bedeutend [6]. Diese Prozesse verknüpfen das Ausmaß des Stressereignisses mit den Energiereserven der Zelle und definieren in Selyes Konzept nach heutigem Verständnis „Stress“ und „strain“ (Abb. 2).

Wichtig in dieser Reaktionskette ist ein negativer Rückkopplungseffekt der Stresshormone und der Signalkaskaden auf die auslösenden neuronalen Prozesse. Diese Rückkopplung ermöglicht nach dem Ende des Stressereignisses eine Wiederherstellung des physiologischen Ausgangszustands oder die Einstellung eines neuen Gleichgewichts [14].

**Abb. 3:**
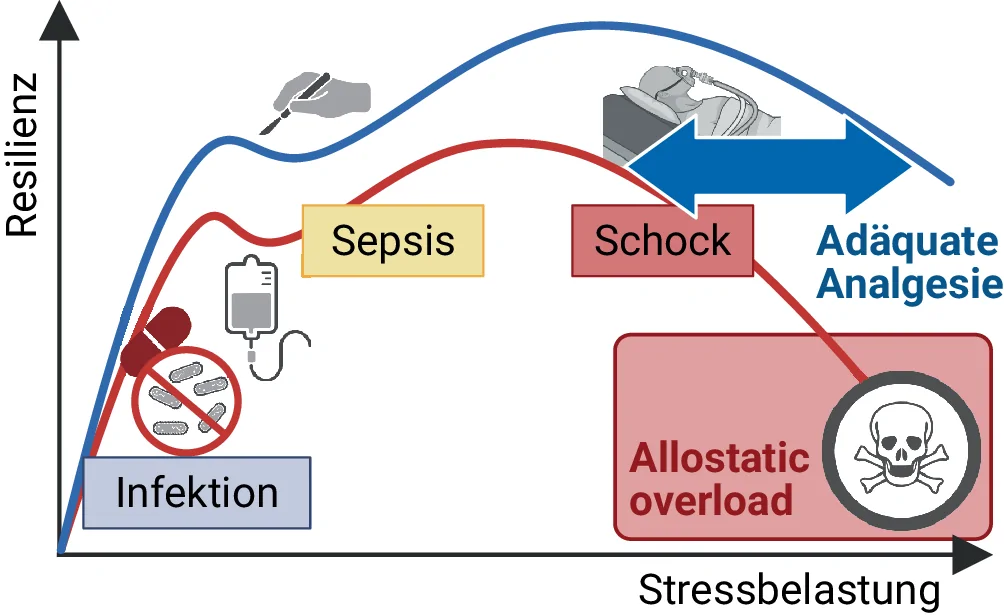
Stellenwert perioperativer medizinischer Maßnahmen und insbesondere einer adäquaten Analgosedierung zur Vermeidung des „allostatic overload“ am Beispiel der chirurgischen Versorgung zur Herdsanierung einer Infektion. Die zellulären Signalkaskaden (Abb. 2) integrieren die verschiedenen Stressoren, wie Pathogenbelastung, Schmerz und Hypoxie zu einer organismischen Antwort, die, wenn der resultierende Stress moderat ist, die Widerstandsfähigkeit des Organismus erhöht. Sind Pathogenstress, Schmerz und Hypoxie in der Summe für den Organismus überwältigend, spricht man im aktuellen Verständnis der Stressbiologie von „allostatic overload“, was zur Überforderung der Reparatur- und Defensivsysteme, Energie- und Organversagen beiträgt.

In der medizinischen Praxis sind die redundanten Reaktionsmuster des Menschen auf Stressoren von hoher Relevanz. Konzeptionell scheint vor dem Hintergrund der Arbeiten Selyes eine Korrektur von Defiziten in der Reaktionsfähigkeit, z. B. im Sinne einer (relativen) Nebenniereninsuffizienz des Organismus auf eine Vielzahl von Stressoren eine Substitutionstherapie mit Stresshormonen, insbesondere Hydrocortison, sinnvoll [8]. Allerdings ist vor dem Hintergrund einer widersprüchlichen Datenlage zur Substitution insbesondere bei Sepsis und septischem Schock [1, 20, 34, 39] die Gabe von Hydrocortison auf ausgewählte klinische Situationen, wie therapierefraktärer septischer Schock und manifeste Nebennierenrindeninsuffizienz, beschränkt. Gleichzeitig kann eine zu tiefe Sedierung mit vollständiger Unterdrückung der Stressantwort in der Intensivmedizin die natürliche Anpassung des Organismus an Stressereignisse, wie Infektion, Operationen und Trauma, mit negativen Effekten auf das Outcome verhindern [12, 41]. Moderne Konzepte der Analgosedierung in der Intensivmedizin werden dem Spannungsfeld aus „Awareness“ und Stress, Delir und Mobilisation durch nuancierte Konzepte mit Fokus auf Analgesie, milder Sedierung und täglichen Sedierungsunterbrechungen gerecht [3]. Ein solches Konzept kann im Kontext von Allostase und Hormese ungünstige Effekte einer zu flachen oder zu tiefen Analgosedierung mit sympathischer adrenerger Aktivierung und hämodynamischer Instabilität verhindern helfen (Abb. 3).

## Dynamik der Stressreaktion

Der Verlauf einer Stressreaktion entscheidet häufig über das Überleben des betroffenen Organismus. Dabei werden Tod oder Überleben nicht nur durch die Stressbelastung, sondern auch durch die Anpassungsfähigkeit oder Resilienz des betroffenen Organismus bestimmt [40]. Die biologische Reaktion auf Stressoren erfolgt aus einem Zustand des relativen Gleichgewichts über eine Phase der Abwehr- und Anpassungsreaktionen (Allostase) zur Einstellung eines neuen Gleichgewichts oder zur Auszehrung des Organismus. Die genannten Reaktionsmuster (Abb. 2) werden wesentlich durch die Energieressourcen der Zellen und Organe des Organismus sowie hiermit verknüpfte Signalwege beeinflusst [6, 37].

Für das Outcome eines Stressereignisses sind die Intensität des auslösenden Stressors, also die Stressbelastung, und die Resilienz (und damit die Energiereserven) in den betroffenen Zellen und Organen gegenüber dem Stressor von Bedeutung [6]. Die überkritische Belastung der Signalkette zwischen auslösendem Signal (Stressor), Mediatoren und Effektormechanismen durch den Stressor wird als „*allostatic overload“* bezeichnet und führt gegebenenfalls zu irreversiblen Schädigungen. Ein klinisch bedeutsames Beispiel stellt das „Takotsubo-Syndrom“ als akute, oft durch extremen emotionalen oder körperlichen Stress ausgelöste Herzmuskelschwäche dar, in dessen Behandlung entsprechend auch β‑Blocker eine bedeutsame Rolle spielen [21]. Zudem kann eine zunehmende stressinduzierte Energiedefizienz bis zur Auszehrung und zum Untergang der betroffenen Zellen und Organe oder dem Tod des Organismus führen [11].

Diese charakteristischen Merkmale von Stressreaktionen werden durch experimentelle Ergebnisse in einer großen Zahl an Modellsystemen und auch durch klinische Beobachtungen unterstützt. Dabei weist die Mehrzahl der Befunde der Stressforschung auf eine prominente Rolle der Stressbelastung bei der Auslösung der spezifischen Stressreaktion hin [4]. Der Versuch, die optimale Stressresilienz von Patienten zu erreichen, muss daher einerseits eine Reduktion den Organismus überlastender Stressreaktionen, z. B. durch Frakturstabilisierung, Herdsanierung bei Infektionen und adäquater Analgosedierung bzw. Narkose, genauso wie eine der Stressreaktion angepasste Ernährungstherapie und Temperaturmanagement umfassen [10, 24]. Umgekehrt ist dieses Konzept geeignet, ungünstige Wirkungen einer zu tiefen Sedierung auf das Outcome von Intensivpatienten zu erklären [42].

## Die Schlüsselfunktion der Stressbelastung

Schon Alltagserfahrungen machen deutlich, dass die Intensität des auslösenden Agens (des Stressors) für die Wirkung des Stressereignisses von großer Bedeutung ist. Während moderate Temperaturabweichungen, geringe mechanische Belastungen oder auch unterkritische Konzentrationen von Toxinen kaum nennenswerten Effekte im Körper auslösen, bewirken überkritische Temperatur‑, Druck- oder Toxinbelastungen häufig irreversible Schädigungen.

Erkennbar besitzen Organismen für die zeitlich und dosisbestimmte Wirkung jeden Stressors einen Toleranzbereich, der vielfach sogar paradoxe also gesundheitsförderliche Wirkungen hervorruft [4]. Im Sinne eingangs genannter Definition und des Konzepts von Sterling erkrankt der Organismus erst bei Überschreiten dieses Schwellenwerts zum „allostatic overload“. Mechanistisch können die entstehenden Schäden durch die begrenzte mechanische Belastbarkeit der Haut, durch eine Überbelastung der Effektkette für die Stressverarbeitung oder auch durch Auszehrung der Energieressourcen entstehen. In der Notfall- und Intensivmedizin ist dabei die synergistische Belastung von Zellen, Organen und des ganzen Organismus durch unterschiedlichste Stressoren von praktisch-medizinischer Bedeutung, da die Effekte dieser Stressoren von den Signaltransduktionskaskaden der Zelle bzw. des Organismus integriert werden (Abb. 2; [5]). So kann z. B. eine nosokomiale Pneumonie nach größerer Chirurgie mit einer erhöhten Gefahr für die Entwicklung eines Organversagens, verglichen mit einer Pneumonie ohne vorhergegangene Operation, einhergehen [13].

**Abb. 4:**
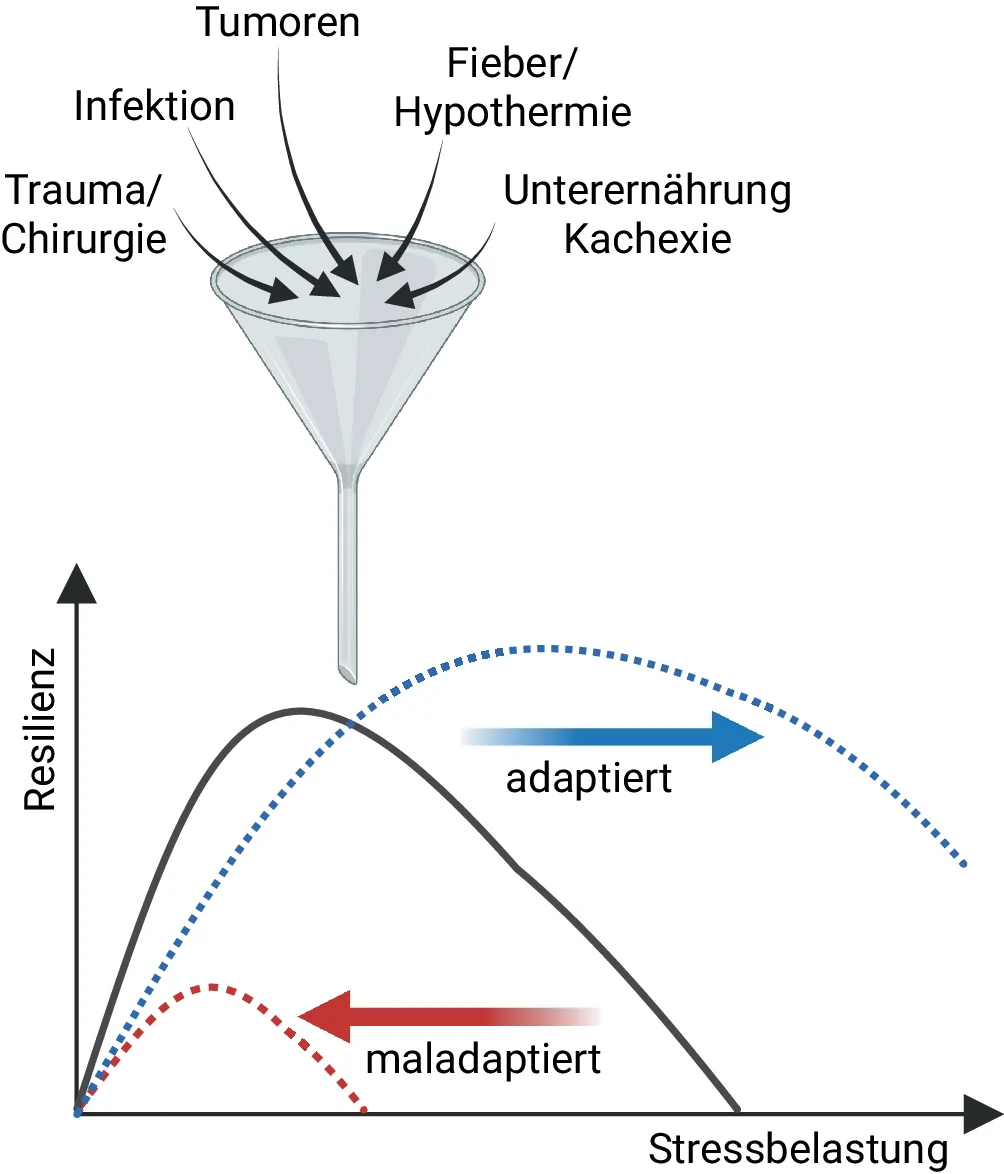
Integration von sequenziellen Stressereignissen und perioperatives Outcome. Im Rahmen schwerer Stressereignisse können Begleitfaktoren beim chirurgischen Patienten, wie Fieber, Kachexie oder Infektion, die Anpassungsreaktion erschweren. Dabei integriert die zelluläre Signaltransduktion das gesamte Ausmaß der Belastungsreaktionen („allostatic load/overload“) zu einer Anpassungsreaktion.

Betrachtet man die individuellen Risiken für Langzeitschäden oder tödliche Folgen durch den Einfluss starker Stressoren, so zeigen experimentelle Untersuchungen als auch medizinische Erkenntnisse deutliche Abweichungen der Stressresilienz von Organismus zu Organismus (Abb. 4). Diese individualspezifischen Unterschiede sind zum einen genetisch bestimmt, entstehen jedoch auch durch unterschiedlichen Kontakt und Erfahrungen mit dem spezifischen Stressor. Während Bewohner unterschiedlicher Klimazonen sich über Generationen an die lokalen Umweltbedingungen anpassten konnten (Adaptation), kann Akklimatisierung auch innerhalb kurzer Zeit stattfinden [23]. Die Anpassung an bestimmte Stressoren kann also offenbar über unterschiedliche genetische und epigenetische Mechanismen trainiert werden [15].

Diese Befunde und Erfahrungen machen deutlich, dass die Effekte von Stress nachhaltig sind und erheblich über die akute Belastung des Organismus hinausgehen. Experimente in vielen Modellsystemen beweisen eine enorme Anpassungsfähigkeit von Organismen gegenüber allen Typen von Stressoren über größere Zeiträume. Grundlage dieser Anpassungsfähigkeit ist ein Prozess namens Hormese. Demnach kann eine niedrige Stressbelastung einen Lern‑, Trainings- und Anpassungseffekt induzieren [9]. Wie in Abb. 4 gezeigt, kann über eine solche hormetische Anpassung durch Konditionierung, d. h. Training, eine deutliche Verringerung der gesundheitsschädlichen Effekte von Stressoren erreicht werden. Die individuellen Unterschiede in der Anpassungsfähigkeit sind also durch Training veränderbar [4, 6, 15].

Die weitreichende Bedeutung dieses Konzepts für die medizinische Praxis soll im nächsten Kapitel am Beispiel der Sepsis – und den aktuellen Konzepten von Resistenz und Toleranz als adäquaten Reaktionsmustern auf eine Infektion – erläutert werden.

## Pathogenstress und Sepsis – Resistenz und Toleranz

Die Pathogenese einer Sepsis im Rahmen einer Infektion kann als ein typisches Beispiel für die Überforderung der Resilienz durch *allostatic overload *aufgefasst werden. Stress, induziert durch mikrobielle Pathogene im Rahmen einer Infektion, kann die Resilienz des betroffenen Patienten durch die kontinuierlich in der Interaktion mit dem eigenen Mikrobiom erhaltene Adaptation überfordern und zu Schädigungen von Zellen und Organen führen. Dabei spielen nach heutigem Verständnis sowohl Über- als auch Unterfunktionen des Immunsystems eine prominente Rolle [2, 16, 26, 30, 32].

Um die Rolle der Stressreaktion im Kontext der Entwicklung einer Sepsis zu verstehen, beschreibt das folgende Kapitel zuerst die spezifischen Merkmale und Effekte von pathogeninduziertem Stress bei Bagatellinfektionen.

Attacken mikrobieller Pathogene stellen ubiquitäre Stressereignisse in der Lebenszeit höherer Organismen dar [33]. Um Schäden durch Pathogenstress zu minimieren, haben diese Organismen zwei Strategien entwickelt, die sich scheinbar ideal ergänzen: Resistenz und Toleranz (im Englischen „disease tolerance“) [22, 35]. Im übertragenen Sinne wirken diese Reaktionen als Schwert und Schutzschild gegen Pathogene. Beide Prozesse tragen zur Resilienz des Organismus im Laufe der Stressreaktion auf Pathogene bei und ergänzen sich gegenseitig [5].

**Resistenzreaktionen** werden im Wesentlichen vom Immunsystem vermittelt. Eingedrungene Pathogene werden durch Immunzellen erkannt und dann durch Phagozytose und verwandte Prozesse vernichtet; damit steht die Reduktion der Keimlast definitionsgemäß im Zentrum der Resistenzreaktionen [19]. Als initialen Schritt der Resistenzreaktionen werden Makrophagen und Granulozyten durch die Pathogene aktiviert. Diese Zellen des angeborenen Immunsystems zerstören dann mit einem toxischen Cocktail aus reaktiven Sauerstoffradikalen und proteolytische Enzymen die pathogenen Mikroorganismen [19]. Dieser potentiell zytotoxische Vorgang, der schon von Paul Ehrlich als „Horror autotoxicus“ im Rahmen der Wirtsantwort auf eine Infektion erkannt wurde, wird durch Zellen des angeborenen und erworbenen Immunsystems und von ihnen ausgeschüttete Zytokine, Chemokine und andere Mediatoren streng kontrolliert.

Im Gegensatz zu Resistenzreaktionen beeinflussen **Toleranzreaktionen** die Zahl der eingedrungenen Pathogene nicht, sondern inaktivieren toxische mikrobielle Produkte oder aktivieren Erhaltungs- und Reparaturreaktionen im befallenen Gewebe bzw. in infektionsortfernen Organen [35]. Diese Regenerationseffekte schließen spezifisch den kontrollierten Abbau von geschädigten Wirtszellen und deren nachfolgende Wiederherstellung ein.

Resistenz- und Toleranzreaktionen auf Pathogenstress sind räumlich und zeitlich eng aufeinander abgestimmt. Die Balance beider Prozesse wird wie bei anderen Stressreaktionen wesentlich durch die Energieladung der beteiligten Zellen bestimmt.

Die aktuelle Forschung unterscheidet zwischen basalen – quasi ererbten – Resistenz- und Toleranzreaktionen und induzierbarer Resistenz und Toleranz [6]. Während die basalen Reaktionen genetisch bestimmt sind, können durch die Konditionierung mit niedrigen Pathogendosen epigenetische Prozesse induziert werden, die eine Verstärkung der Resistenzreaktionen („Training“) und auch der Toleranzreaktionen für einen längeren Zeitraum fixieren [6].

Die Induzierbarkeit der Resistenz- und Toleranzreaktionen hängt stark von der Pathogenstressbelastung ab und folgt damit dem Hormeseprinzip. Experimentelle Befunde zeigen, dass niedrige Pathogenlasten zuerst Resistenzreaktionen aktivieren. Bei höherer Pathogenbelastung folgt dann zunehmend Pathogentoleranz als zweites Element der hormetischen Anpassung [4, 6]. Extrem hohe Pathogenlasten schließlich überfordern die basalen und adaptiven Resistenz- und Toleranzreaktionen und damit die Resilienz des Organismus gegenüber dem Pathogenstress mit der Folge einer Überschreitung der Reserven, im englischen Schrifttum „*allostatic overload*“, mit der Folge einer Organdysfunktion [11].

## *Allostatic overload* und Auszehrung in der Sepsis

Im Krankheitsbild der Sepsis dominieren Fehlfunktionen von Resistenz und Toleranz.

Akuter Pathogenstress bei Sepsis kann zu unkontrollierter Hyperinflammation und zu raschem Energieverbrauch führen oder zu Immunparalyse und Auszehrung. Beides mündet in schweren Fällen in *allostatic overload* und Organdysfunktion [11]. Beide Prozesse sind mit zunehmenden Schäden des parenchymalen Gewebes verbunden. Während beim Phänotyp der Hyperinflammation aktivierte Immunzellen durch die Freisetzung von proteolytischen Enzymen oder reaktiven Sauerstoffradikalen das Umgebungsgewebe zerstören, sind es die pathogenen Mikroorganismen, die sich ungehindert während der Immunparalyse ausbreiten und dann Organschäden bewirken [26].

Die von Selye beobachteten Redundanzen der Reaktionen von höheren Organismen auf verschieden Formen von Stress beziehen die Resilienzreaktionen auf Pathogenstress mit ein. Stressoren wie Hitze, Kälte und Toxine konkurrieren mit Pathogenen um überlappende Ressourcen und Signalwege [5, 38]. In der Entstehung von *allostatic overload* beziehungsweise energetisch bestimmter Auszehrung bestimmen Synergieeffekte zwischen den einzelnen Stressoren die Morbidität und Mortalität des Stressereignisses (Abb. 4). Eine zusammenfassende Übersicht über die Effekte ausgewählter Stressoren, adaptive Reaktionen, maladaptive Reaktionen, die entsprechenden klinischen Korrelate und therapeutische Konsequenzen zeigt Tab. 1.

**Tab. 1:** Beispiele bedeutsamer Stressoren und (mal-)adaptiver Reaktionen sowie klinische Korrelate und therapeutische Konzepte

Stressor	Adaptive Reaktion	Maladaptive Reaktion	Klinisches Korrelat	Therapeutische Konzepte
Infektion	Resistenz- und Toleranz-Reaktionen, Regeneration	Hyperinflammation	Septischer Schock	IL-1-RA [17]
		Immunsuppression	Opportunistische Infektionen	γ‑IFN [17]
Gewebetrauma Mechanischer Stress/Blutung	Resistenz- und Toleranzreaktionen, Regeneration	SIRS	–	Minimalinvasive Chirurgie Analgosedierung Restriktive Transfusion
		Immunsuppression		
Hypothermie	Thermoregulation, Vasokonstriktion, Muskelzittern	–	„Shivering“	Wärmemanagement

*γIFN* gamma-Interferon; *IL-1-RA* Interleukin-1 receptor antagonist; *SIRS* Systemic Inflammatory Response Syndrome

### Infobox Biografische Vignette von Hans Selye *(1907–1982)*, dem Begründer der modernen Stressforschung

Hans Hugo Bruno Selye wurde in Wien geboren, wuchs in Österreich-Ungarn auf und wirkte später als translationaler Mediziner und Biochemiker in Prag, Rom, Paris und schließlich an der McGill University Montréal, Kanada. Während seiner Forschungen an der McGill University beobachtete Selye, dass Ratten auf unterschiedlichste schädliche Reize (Kälte, Medikamente, Verletzungen) unspezifisch mit denselben körperlichen Symptomen reagierten, und zwar klinisch mit Vergrößerung der Nebennierenrinde, Schrumpfung des Thymus und Magengeschwüren.

Selye prägte den Begriff „Stress“ und definierte ihn als die „unspezifische Reaktion des Organismus auf jede Art von Anforderung“ oder als *Allgemeines Anpassungssyndrom*, welches Stress in drei Phasen beschreibt: Alarmreaktion, Widerstandsphase und Erschöpfungsphase. Selye unterschied zwischen schädlichem „Distress“ und positivem „Eustress“. Er verfasste über 1700 wissenschaftliche Artikel und 39 Bücher, darunter The *Stress of Life* (1956) [43] und seine Autobiografie *Stress – mein Leben* [44]. Selye wurde mehrfach für den Nobelpreis nominiert. Selye verstarb 1982 in Montréal.

## Fazit für die Praxis

- Die Fähigkeit zur Stressverarbeitung bestimmt wesentlich den Gesundheitszustand des Organismus. Die Gabe von Stressmediatoren, wie Adrenalin oder Cortison, kann daher eine therapeutische Wirkung entfalten und so Patienten für besondere Belastungen konditionieren .
- Wegen der begrenzten Kapazität der Allostase muss eine Überlastung der Stressverarbeitungsprozesse vermieden werden. So ist z. B. während einer Operation zur Herdsanierung zusätzlicher Stress durch suboptimale Temperaturen, Schmerz oder unangepasste Zufuhr von Nährstoffen ungünstig für das Outcome.
- Die Wirkung von Stressoren auf den Organismus hängt stark von deren Intensität ab. Aktuelle Studien dokumentieren , dass die Stressantwort durch eine nichtlineare Dosis-Wirkung-Beziehung charakterisiert ist (Hormese). Im Falle von Infektionen bestimmen Pathogenlast und Verfügbarkeit von Energie über physiologische oder pathologische Effekte der Abwehrreaktionen.
- Diese Betrachtungsweisen der „allgemeinen Anpassungsreaktion“, vor 90 Jahren durch Hans Selye formuliert, sind geeignet, im Kontext neuerer Erkenntnisse unser Verständnis zur Entwicklung eines Organversagens im Rahmen einer Infektion fundamental weiterzuentwickeln.

## Data Availability

Alle dieser Arbeit zugrunde liegenden Daten sind in diesem Artikel enthalten.
